# Anatomical morphometry for Cricothyrotomy puncture and incision

**DOI:** 10.1186/s12893-023-02100-9

**Published:** 2023-07-12

**Authors:** Kaiji Suzuki, Naohito Yambe, Kentaro Hojo, Yasunori Komatsu, Masamitsu Serikawa, Akinobu Usami

**Affiliations:** 1 Department of Oral Functional Anatomy, Graduate School of Dentistry, Ohu University, Koriyama, Japan; 2 Community Medicine Support Dentistry, Ohu University Hospital, Koriyama, Japan; 3 Department of Oral Anesthesia, School of Dentistry, Ohu University, Koriyama, Japan; 4 Department of Morphological Biology, School of Dentistry, Ohu University, Koriyama, Japan

**Keywords:** Cricothyroid ligament, Airway management, Stature estimation, Cricothyrotomy

## Abstract

**Purpose:**

Emergency surgical airway securing techniques include cricothyrotomy, puncture, and incision. While the instruments used for these methods vary in size, no index of laryngeal morphology exists to guide instrument selection. Therefore, we measured the morphology of the cricothyroid ligament in Japanese individuals and assessed its correlations with height.

**Methods:**

This retrospective study used 61 anatomical practice specimens. The cricothyroid ligament of the laryngeal area was dissected, and a frontal image was recorded. Next, images of the midsagittal sections of the larynx and trachea were recorded. The width and height of the cricothyroid ligament were measured from the frontal images, and the depth of the larynx and the angle to the lower edge of the cricothyroid plate were measured from the mid-sagittal cross-sectional images. The height was estimated from the tibial lengths of the specimens and statistically analyzed for correlations.

**Results:**

The width and depth were significantly greater in males. Overall, there was a slight correlation between the results of each laryngeal measurement and estimated height for all items.

**Conclusion:**

The morphology of cricothyrotomy revealed that the width and depth of the laryngeal area varied according to sex. Moreover, the results also showed a correlation with the estimated height. Thus, it is important to predict the morphology of the laryngeal area and cricothyroid ligament by considering factors such as patient sex, weight, and height.

## Background

Surgical airway clearance techniques include puncture and the incision cricothyrotomy [1–3], and tracheotomy. In recent years, the instruments for these techniques have advanced remarkably [4]. The two main methods of cricothyrotomy are the Seldinger method and the direct puncture method. Many reports have demonstrated the effectiveness of the cricothyrotomy instruments used in these methods and have described the changes in procedure time and complications with practical training. Various simulation methods have been used for these comparisons. The samples for practical training vary from donated bodies [5–7] to simulators [8] and animals such as sheep and pigs [9–11].

However, treatment for trauma or bleeding in the maxillofacial region may be necessary before a patient is transported to the hospital [12, 8]. Emergency airway clearance by puncture of the cricothyroid ligament requires experience, and training is important for inexperienced personnel [13]. The accurate identification of the cricothyroid ligament is difficult in emergencies, and there have been reports of damage to the tracheobronchial site, unsuccessful puncture, and incorrect insertion [6, 7]. Therefore, the use of ultrasonography and palpation [14, 15] is recommended.

To address these challenges, many training facilities in Japan provide cadaver surgical training (CST) [16]. However, simulator training is still needed, as cadaver availability is limited to certain facilities and environments. Clinical experience is also necessary because it does not increase the experience value by itself; however, there are few reports on the anatomical morphology of the perilaryngeal anatomy associated with cricothyrotomy as a guideline for this procedure.

The bodies used in this study were preserved in various postures and their height were not recorded. Therefore, we measured the perilaryngeal morphology of Japanese donors; estimated their height from tibia length; and searched for relationships between sex, estimated height, and cricothyroid ligament morphology.

## Materials and methods

The samples were 61 Japanese adult donors (30 males and 31 females, age 82.9 ± 10.6 years) who had been preserved and treated. This study was approved by the Ethical Review Committee of Ohu University (Approval No. 234).

**Fig. 1:**
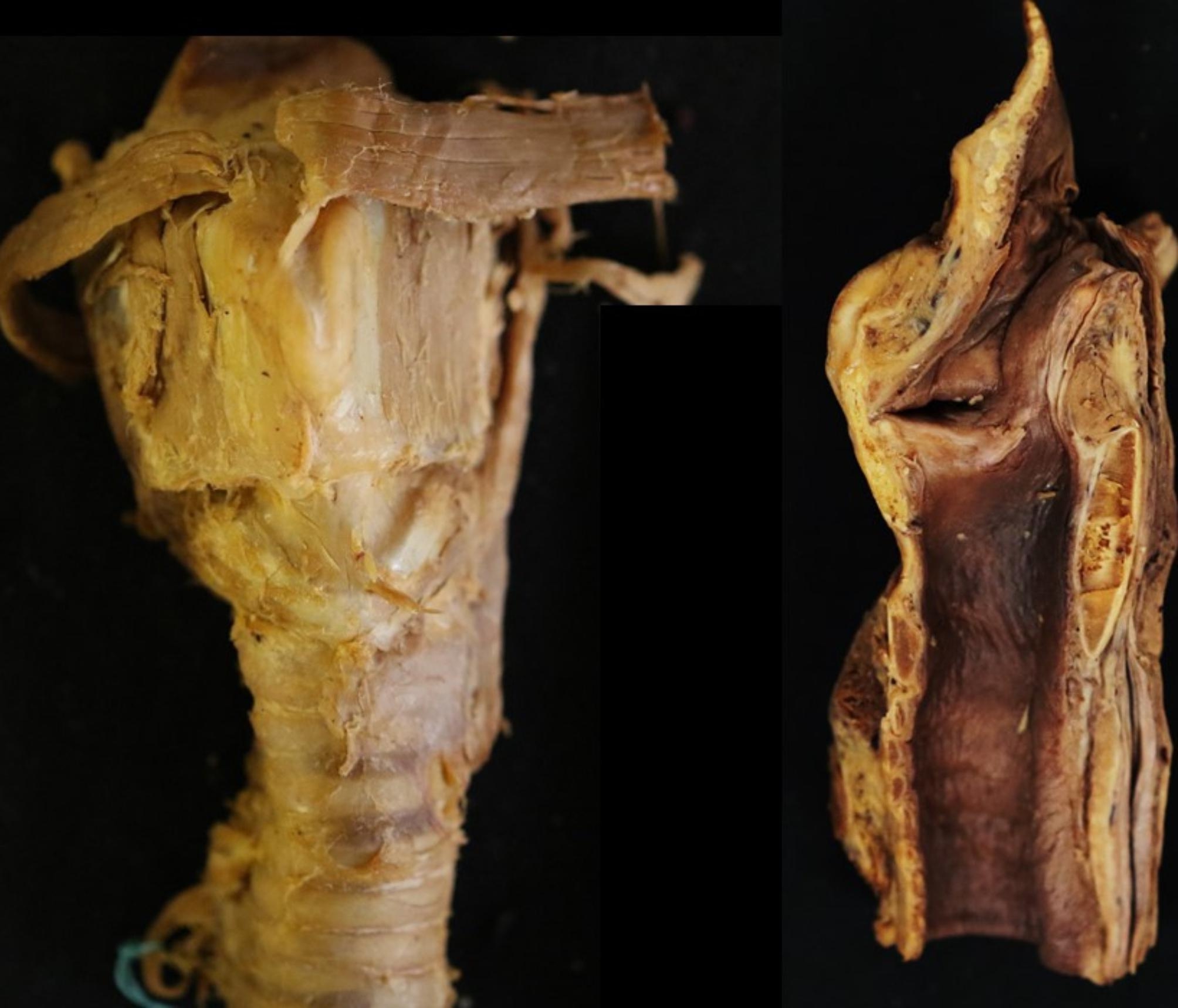
Sample images. Left: Frontal images. Right: Mid-sagittal sectional image

After making skin incisions of the neck, the perilaryngeal area was removed in a single lump below the hyoid bone to the tracheal cartilage, with the platysma, infrahyoid muscles, and cricothyroid muscles detached. After removing the sublingual and cricothyroid muscles, the cricothyroid ligament was photographed with a digital camera (EOS 7D Mark II, Canon) from the frontal direction for morphometric measurements of the width and height. The larynx was then cut in half in the midline along the trachea and photographed from a direction perpendicular to the midline sagittal section (Fig. 1).

**Fig. 2:**
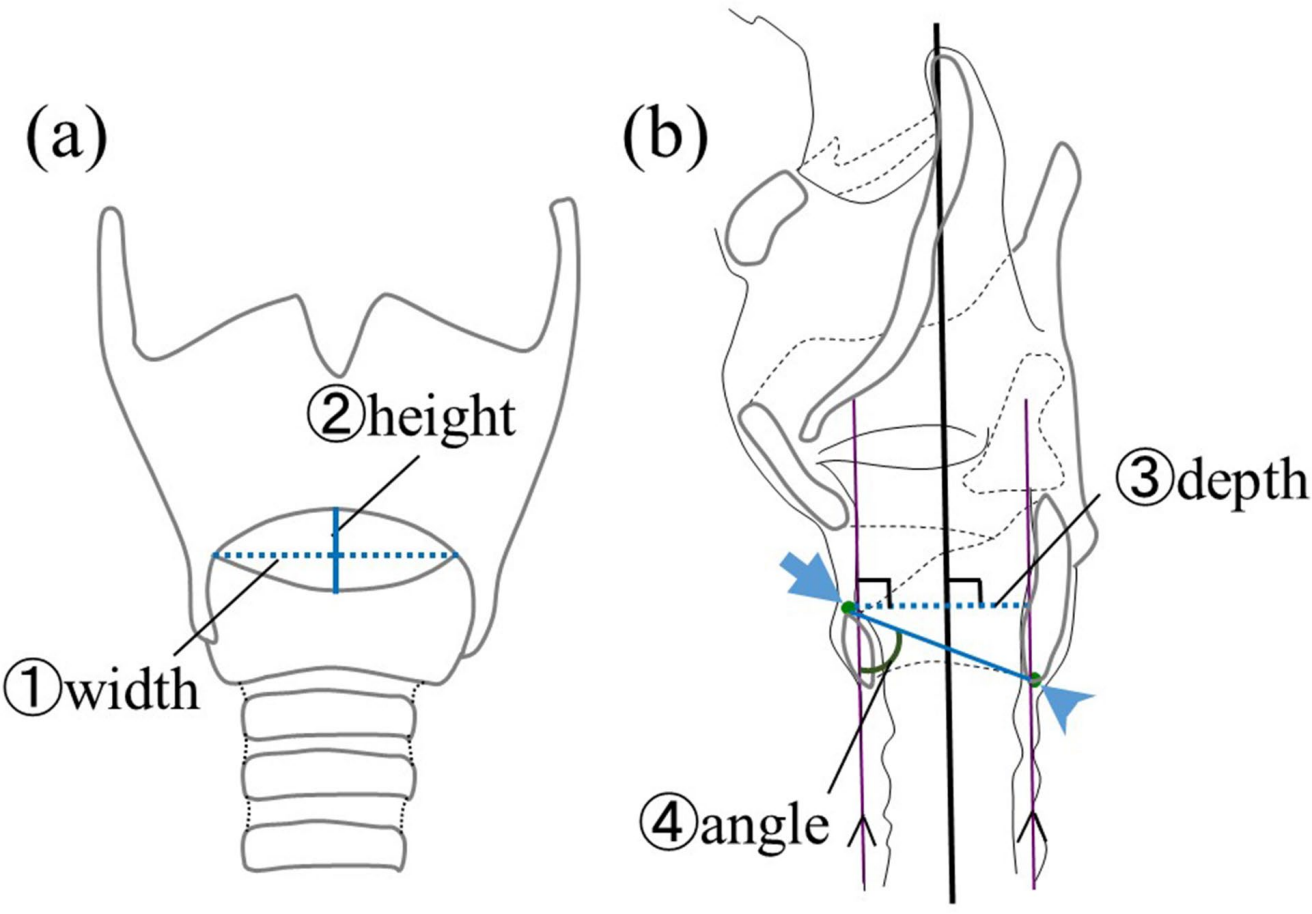
Measured items. **(a)** Frontal view. Dotted line: width diameter, solid line: height. **(b)** Midsagittal cross-sectional image. Bold line: long axis of the trachea, arrow: anterior superior margin of the cricoid cartilage, arrowhead: inferior end of the cricoid cartilage plate

The following items were measured in this study: ① the width and ② height of the cricothyroid ligament and ③ the depth and ④ angle of the inner laryngeal. The width and height of the cricothyroid ligament in the frontal image were the maximum vertical and lateral lengths of the cricothyroid ligament. The depth and angle were measured in the midsagittal section images. The depth was measured from the anterior superior margin of the cricoid cartilage to the anterior margin of the cricoid cartilage plate in a straight line perpendicular to the long axis of the trachea. This angle was defined as the angle between the line segment connecting the anterior superior border of the cricoid cartilage to the inferior edge of the cricoid cartilage plate made with the long axis of the trachea (Fig. 2).

Each item was measured using the publicly available ImageJ software [17]. Tibia length was measured in all samples, and height was estimated using Fujii’s height estimation formula [18] (Table 1).

**Table 1:** Regression linear equation for height estimation from tibia length by Fujii^19)^. X: tibia length (mm), Y: estimated height (mm)

Males	Left: Y = 2.36X + 775.42
	Right: Y = 2.47X + 739.99
Females	Left: Y = 2.34X + 737.54
	Right: Y = 2.20X + 778.71

For statistical analysis, the Mann–Whitney U test was performed to test for significant sex differences in the measurement results. Correlation coefficients between each measured item and the estimated height were also obtained.

## Results

The cricothyroid ligament measured an average of 17.7 mm in width (19.8 mm in males and 16.0 mm in females). The mean height was 8.1 mm (8.4 mm for males and 7.8 mm for females). The mean depth was 19.9 mm (21.9 mm for males and 18.0 mm for females). The average angle was 66.2° (66.3° in males and 66.1° in females) (Table 2).

**Table 2:** Correlation coefficients between estimated height and cricothyroid ligament measurements by sex

	Overall	Male	Female
**Estimated height (cm)**	154.0 ± 7.5	159.4 ± 6.0	148.0 ± 7.5
**Width (mm)**			
Mean ± SD	17.7 ± 2.9	19.8 ± 2.5*	16.0 ± 2.9*
correlation coefficient	0.49	-0.01	-0.06
**Height (mm)**			
Mean ± SD	8.1 ± 2.0	8.4 ± 2.3	7.8 ± 2.0
correlation coefficient	0.3	0.41	0.01
**Depth (mm)**			
Mean ± SD	19.9 ± 2.7	21.9 ± 2.4*	18.0 ± 2.7*
correlation coefficient	0.6	0.24	-0.02
**Angle (°)**			
Mean ± SD	66.2 ± 7.4	66.3 ± 7.9	66.1 ± 7.4
Correlation coefficients	-0.21	-0.47	-0.09

*:significant difference (p < 0.05)

Sex differences were observed in width and depth. The average estimated height obtained from Fujii’s regression line was 154.0 cm (159.4 cm for males and 148.0 cm for females).

**Fig. 3:**
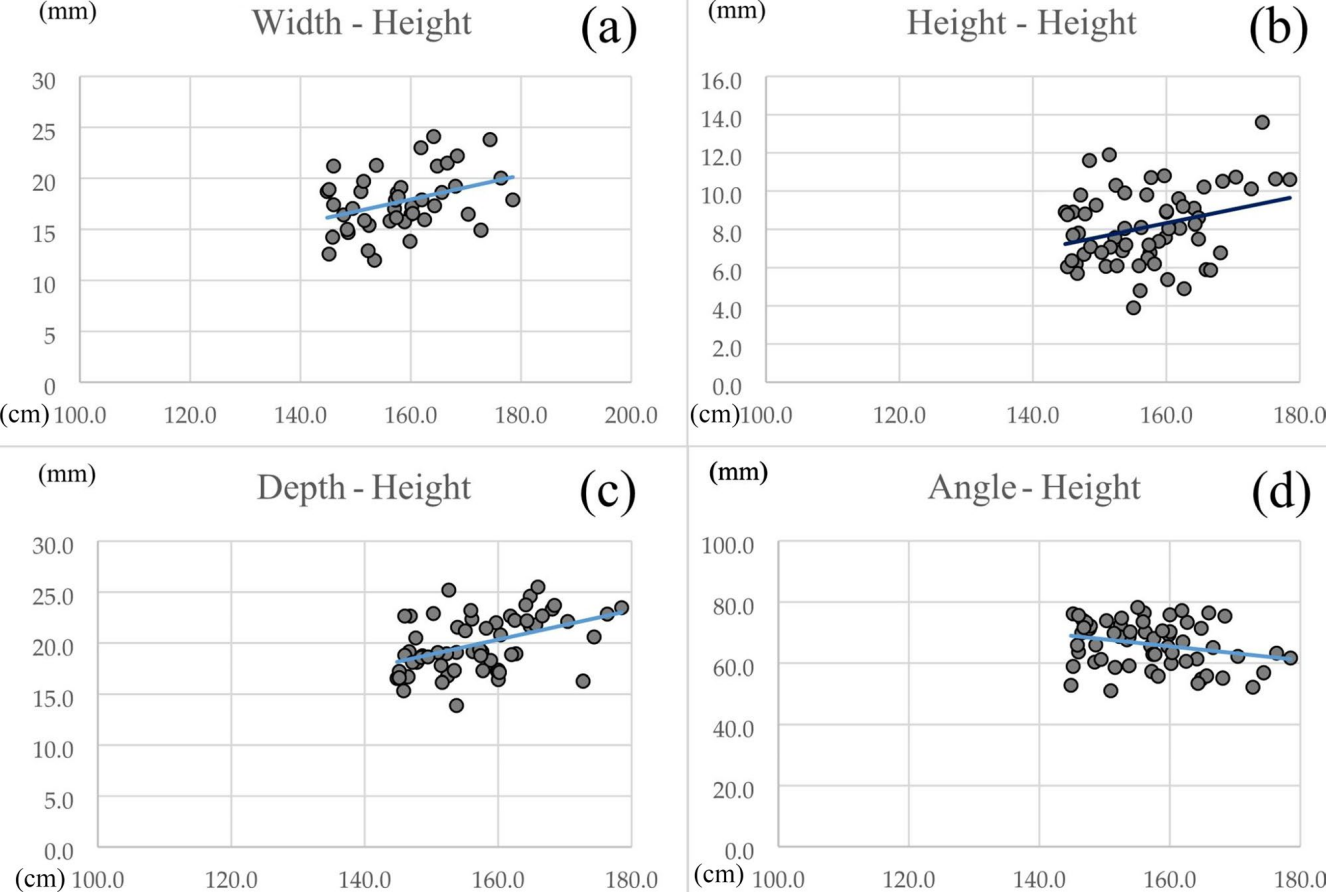
Correlations of cricothyroid ligament measurements with estimated height. **(a)** Correlation between the width and estimated height. **(b)** Correlation between height and estimated height. **(c)** Correlation between depth and estimated height. **(d)** Correlation between angle and estimated height

The correlations between the estimated height and cricothyroid ligament measurements were 0.49 for width, 0.30 for height, 0.60 for depth, and − 0.21 for angle. A correlation was observed for depth, while weak correlations were observed for all the other measures (Fig. 3).

## Discussion

Cricothyrotomy is a prehospital life-saving method used for the immediate resolution in case in which intubation and oxygenation are not possible [19, 20]. In recent years, cricothyrotomy for airway clearance has become available for a variety of devices, such as Melker and QuickTrach II [4]. The two main methods of cricothyrotomy are the Seldinger and direct puncture methods. Many reports have described the effectiveness of the equipment used for this procedure as well as the need for practical training and the risks of complications [5–11, 21, 22].

However, the difficulty in performing cricothyroid ligament surgery in the emergency setting is that the incision position cannot be accurately determined [1]. Therefore, complications such as injury to the tracheobronchial site, puncture failure, and incorrect insertion may occur. Although cricothyrotomy is a generally safe, convenient, and rapid procedure, the risk of cartilage damage has been reported [6, 7]. Kress et al. suggested that the skin incision should be made horizontally, 1–2 cm in length and that the instrumentation should be directed downward at 30–40 degrees [3]. Schober et al. reported a novel shear for cricothyrotomy, with a metal stopper 19 mm from the tip of the blade to limit the depth. In addition, it is possible to expand by 15 mm in the horizontal direction using this method [7]. However, there are no reports on the relationship between patient body size, skin incision length and depth, and the average cricothyroid ligament size. While Fennesy et al. reported the angle of the neck and position of the cricothyroid ligament at the time of puncture, no reports have described the size of the cricothyroid ligament relative to body size [23].

An additional problem is that the terminology used to describe the cricothyrotomy procedure is not consistent. That is, the cricothyroid and cricopharyngeal membranes are used as synonyms for the elastic cone, and the median portion of the elastic cone is sometimes called the anterior cricothyroid ligament or the median cricothyroid ligament [24–26].

To obtain an index for proper instrument selection in cricothyrotomy, we measured the morphology of the cricothyroid ligament in adult Japanese donors. The mean width of the cricothyroid ligament was 17.7 ± 2.9 mm and the mean height was 8.1 ± 2.0 mm. Dover et al. removed cricothyroid ligament width and height (at the widest point) measures of 10.9 mm and 10.4 mm, respectively [27]. The difference in width is likely because Dover et al. flipped only the sublingual muscle, whereas the present study also flipped the cricothyroid muscle and measured the morphology of the cartilage around the cricothyroid ligament itself. While the height measurements were similar between the studies, the smaller values in the Japanese donors suggest the possible effects of the estimated height and race on the results. The average depth measurements in this study were 19.9 mm. The stopper of the shears proposed by Schober et al. [7] was at the 19 mm position, which could damage the cricoid cartilage plate on the posterior wall of the larynx if used in Japan. In the present study, the angle between the line segment connecting the anterior superior margin of the cricoid cartilage and the inferior end of the posterior cricoid cartilage plate with the long axis of the trachea was 66.2°. While Kress et al. instructed that the instruments be inserted 30–40° downward [3], in this range, the absence of the cricoid cartilage plate posteriorly increased the risk of penetration into the tracheobronchial-like area. Dover et al. recommended the use of a tracheal tube 9–10 mm in diameter for cricothyrotomy [27]. The measurements in this study differed significantly in width and depth results, with smaller values in women. In other words, to improve safety, the instrument used for cricothyrotomy must be appropriately sized for individual larynxes. For instance, a narrower cannula must be used to pass the cricothyroid membrane in Japanese patients of short height.

Complications have also been reported in cricothyrotomy, including injury to the tracheobronchial area and damage to the cricoid and thyroid cartilage [6, 11, 19, 20]. As cricothyrotomy requires a longer procedure time in inexperienced clinicians, prior practical training is important [5, 8–11, 21, 22]. In recent years, many training facilities in Japan have provided CST [16]; however, the need for simulator training remains because of the limitations of the facilities and other environments. Various condition settings have been made to simulate cricothyrotomy methods [5–11]. The samples for practical training vary among donated bodies [5–7], simulators [8], and animals such as sheep and pigs [9–11]. When animals are used for ethical and practical reasons, differences in soft tissue thickness and other factors may affect work time [10]. Therefore, the relationship between the instruments used and the cricothyroid ligament size must also be examined. Some reports have shown that computed tomography (CT) observation does not reveal the presence of blood vessels anterior to the cricothyroid ligament [22]. However, a retrospective study reported bleeding as the main complication [28]. Because there is no bleeding in the donor-based training method, the actual procedure is not affected by vascular injury [6]. Therefore, clinical experience is required for emergency airway management by puncturing the cricothyroid ligament, and training is important for inexperienced individuals [13]. Complications are particularly likely to occur when the patient’s neck is thick and landmarks are difficult to palpate [6]. The average estimated height of the participants in this study was 154.0 cm, as determined from the regression line for estimating height in the Japanese population. The estimated height and cricothyroid ligament measurements were correlated with depth and weakly correlated with all other measurements. Thus, not only body size but also patient height may affect the risk of injury in cricothyrotomy.

Limitations.

As this was a cadaver study, no data on height, weight, or BMI of the donor bodies were available. In addition, the donors were older adults and their physiques biased accordingly. Moreover, the soft tissue may have been slightly deformed due to preservative treatment.

## Conclusion

It is important to determine the correct incision size to ensure a safe cricothyrotomy. The present measurements of the cricothyroid ligament in the Japanese showed sex differences in width and depth, as well as smaller values compared to previous reports from overseas. The correlation between height and laryngeal measurement values suggested the important need for the preoperative evaluation of height and physique in emergency cricothyrotomy.

## Data Availability

The datasets used and/or analyzed during the current study available from the corresponding author on reasonable request.
